# Hypercalcemia in a Patient With Spinal Tuberculosis and Aspergillus Co‐Infection: A Case‐Report

**DOI:** 10.1002/iid3.70508

**Published:** 2026-09-09

**Authors:** Negin Parsa, Amirpasha Amlelshahbaz, Hamidreza Soltani

**Affiliations:** ^1^ Student Research Committee Shahid Sadoughi University of Medical Sciences and Health Services Yazd Iran; ^2^ Department of Radiology Shahid Sadoughi University of Medical Sciences and Health Services Yazd Iran; ^3^ Department of Internal Medicine Rheumatology, Shahid Sadoughi University of Medical Sciences and Health Services Yazd Iran

**Keywords:** case report, hypercalcemia, paraplegia, prolonged backpain, spinal tuberculosis, tuberculosis

## Abstract

**Background:**

Tuberculosis (TB), one of the leading infectious causes of mortality worldwide, may disseminate hematogenously and result in extrapulmonary manifestations. Spinal tuberculosis (ST) is an uncommon but serious form of extrapulmonary TB. In addition, TB may predispose patient to opportunistic infections, including Aspergillus species.

**Case Presentation:**

A 62‐year‐old man presented with chronic back pain and sudden paraplegia. High‐resolution computed tomography (HRCT) of the chest demonstrated findings suggestive of miliary TB, which was supported by a strongly positive purified protein derivative (PPD) test and a positive sputum smears for acid‐fast bacilli. Anti‐TB therapy was subsequently initiated. Aspiration of an epidural abscess revealed a positive polymerase chain reaction (PCR) result for *Mycobacterium tuberculosis*, while histopathological examination demonstrated septate hyaline hyphae suggestive of Aspergillus species. During hospitalization, the patient developed recurrent fever, agitation, and progression of pulmonary lesions. In conjunction with a positive serum galactomannan assay, these findings raised suspicion for concomitant aspergillosis infection, prompting initiation antifungal therapy with amphotericin B and voriconazole. A major clinical challenge was severe hypercalcemia, which progressed despite intravenous hydration and calcitonin therapy, reaching a peak serum calcium level of 18.01 mg/dl. Subsequent treatment with pamidronate resulted in gradual normalization of serum calcium levels.

**Conclusion:**

This case highlights the diagnostic and therapeutic challenges associated with spinal tuberculosis, probable Aspergillus co‐infection, and severe treatment‐resistant hypercalcemia. Clinicians should consider opportunistic fungal infections and metabolic complications when evaluating patients with disseminated TB.

## 1 Introduction and Literature Review

Tuberculosis (TB) ranks as the second leading infectious cause of death globally [1]. Spinal tuberculosis (ST), also referred as Pott's disease, vertebral tuberculosis, tuberculous spondylitis, or tuberculous spondylodiscitis, accounts for less than 1% of all TB cases and is the most common form of skeletal tuberculosis, comprising approximately 50% of all skeletal cases [2, 3].

ST is most commonly caused by secondary hematogenous spread of *Mycobacterium tuberculosis (MTB)* from either a quiescent or active pulmonary focus [4]. Less frequently, it may result from contiguous extension or lymphatic dissemination from pleural diseases [4].

In immunocompetent individuals, granuloma formation in response to infection helps limit the replication and dissemination of MTB [5]. In contrast, immunocompromised individuals demonstrate a reduced capacity to effectively contain the pathogen, placing them at a substantially higher risk of developing disseminated TB. Additional factors, including advanced age and female sex, have also been associated with an increased likelihood of extrapulmonary TB involvement [4].

The clinical manifestations of ST are often nonspecific. Notably, ST may occur in patients with normal chest radiographs and negative sputum smears; therefore, delayed diagnosis of extrapulmonary disease is common [6].

The association between pulmonary TB and pulmonary fungal co‐infections has recently attracted considerable attention, as either condition may occur secondary to the other [7]. Chronic pulmonary aspergillosis (CPA) is a well‐established complication of pulmonary TB [8]. Cavitary lesions resulting from TB, together with immunosuppression, appear to predisposes patients to Aspergillus infection [7, 9]. Among Aspergillus species, *A. fumigatus* is responsible for some of the most devastating pulmonary infections in terms of morbidity and mortality; however, in the context of TB, it may progress insidiously and remain clinically silent for years [7, 10].

Hypercalcemia has been reported as a common complication of TB, including both pulmonary and extrapulmonary forms [7]. The reported prevalence varies, ranging from approximately 2.3% to as high as 10%–48% in different studies [11]. The precise reason for this variability is uncertain [1, 11].

An increase in serum 1,25‐dihydroxyvitamin D_3_ [1,25 (OH)_2_ D_3_] concentrations resulting from external production by activated macrophages in granulomatous diseases such as TB, sarcoidosis, and certain fungal infections is presumed to be the principal mechanism underlying hypercalcemia [11, 12].

The patient described in this report presented with severe and nonspecific clinical manifestations, posing substantial diagnostic challenges. In addition, he developed abrupt and marked hypercalcemia that was poorly responsive to initial therapy. The rarity of clinical presentation, together with the diagnostic and therapeutic challenges encountered during management, prompted us to report this case.

### 1.1 Case Report

A 64‐year‐old male with unremarkable past medical history was admitted to Shahid Rahnemoon hospital, Yazd, Iran. He presented with progressive back pain of 3 months' duration, localized to the bilateral lumbosacral region and radiating to both lower limbs. Almost a week prior, he suddenly lost the ability of walking. He also reported nocturnal pain, fever, loss of appetite, and significant weight loss of approximately 10 kilograms over the preceding 3 months. Additionally, he had experienced complete urinary incontinency in the past 2 days.

The patient was a smoker and mentioned addiction to Methadone tablets. He denied any other substance or medication use. He was born and raised in Afghanistan and had recently migrated to Iran.

Physical examination revealed diminished knee reflexes and marked motor weakness, with muscle strength graded 1/5 in the upper extremities and 2/5 in the lower extremities. The right elbow was painful on active movement and demonstrated a reduced range of motion. The straight‐leg‐raise test was positive bilaterally. Bilateral digital clubbing was noted. No sensory deficits were identified. Lumbar tenderness was present at the of L_4_–L_5_ level. There were no overlying signs of inflammation, such as erythema or swelling.

Electromyography‐nerve conduction velocity studies were initially performed. The findings raised suspicion for an underlying paraneoplastic process and demonstrated chronic bilateral moderate‐to‐severe L_5_–S_1_ radiculopathy as well as moderate C_5_–C_6_ root involvement. No active denervation was detected.

**Figure 1:**
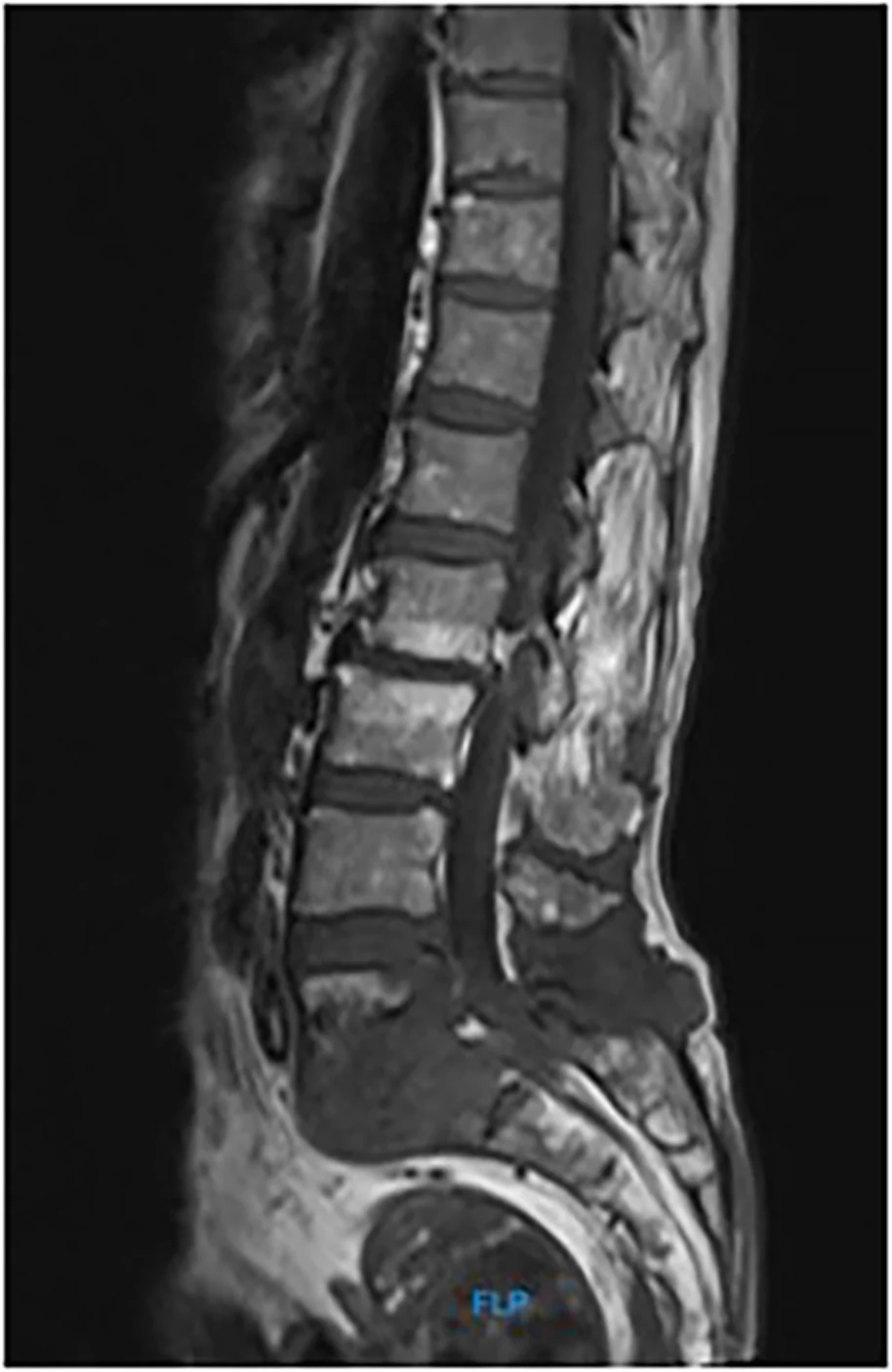
Non‐contrast lumbosacral MRI demonstrating bone marrow edema involving the L5, S1, and S2 vertebral bodies, with destruction of the L5–S1 endplates and associated discitis. The inflammatory process extends to the posterior spinal elements and adjucent paravertebral muscles.

Given the patient's sudden motor deterioration and evidence of lower motor neuron dysfunction, magnetic resonance imaging (MRI) of the central nervous system was obtained. Brain MRI findings were unremarkable. However, cervical and lumbar MRI revealed asymmetric osteophyte‐disc complexes at C_3_–C_4_ and C_4_–C_5_ levels, along with diffuse disc bulging at the C_5_–C_6_ and C_6_–C_7_ levels, causing thecal sac compression. Representative MRI findings are shown in Figure 1.

An epidural abscess at the L_5_–S_1_ was identified, causing severe spinal canal stenosis. In addition, a paravertebral abscess was observed. The absence of local inflammatory signs and symptoms was suggestive of a cold abscess. MRI also demonstrated irregularity and destruction of the vertebral endplates and anterior portions of the vertebral bodies, accompanied by bone marrow edema at the S_1_–S_2_ level, findings consistent with spondylodiscitis.

The severe neural compression associated with the epidural abscess, together with the abrupt motor deterioration, warranted urgent intervention. Surgical decompression and abscess drainage were recommended; however, the patient declined surgical treatment and opted for conservative management.

**Figure 2:**
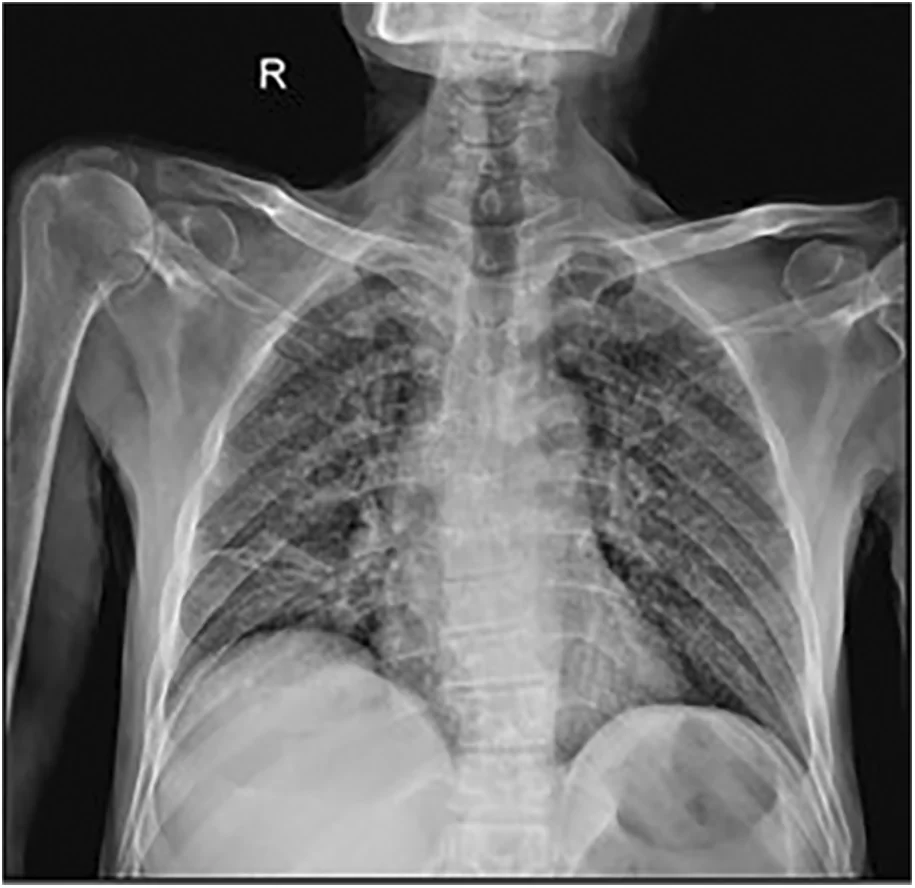
Chest radiograph obtained on the first day of admission demonstrating bilateral diffuse patchy reticulonodular opacities, predominantly involving the upper lobes and perihilar regions.

Given the characteristic MRI findings, the patient's social history, and his recent immigration from a TB‐endemic region, TB was considered a leading differential diagnosis. Accordingly, a chest radiograph was obtained and revealed bilateral diffuse patchy pulmonary opacities suggestive of active pulmonary TB (Figure 2).

**Figure 3:**
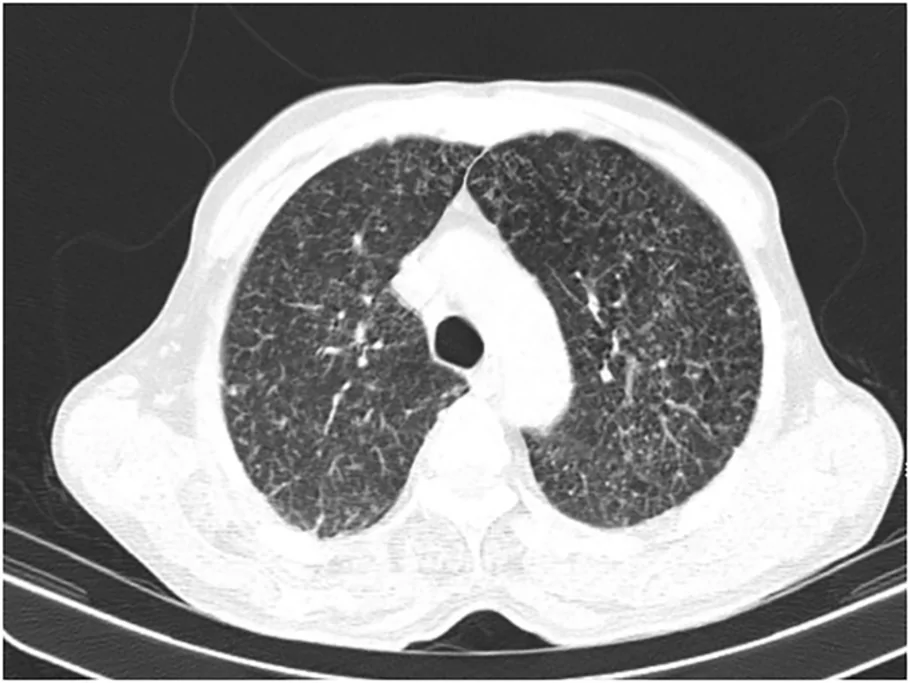
High‐resolution computed tomography (HRCT) of the chest demonstrating multiple diffuse bilateral micronodules, consistent with disseminated pulmonary tuberculosis.

To further characterize the pulmonary abnormalities, high‐resolution computed tomography (HRCT) of the chest was performed. Because there was no clinical suspicion of vascular pathology or mediastinal disease, intravenous contrast was not administered. HRCT demonstrated diffuse micronodular and interstitial pulmonary infiltrates consistent with miliary TB (Figure 3).

Further investigations strongly supported the diagnosis. The purified protein derivative (PPD) skin test showed an induration of 30 mm, and sputum smear microscopy was positive for acid‐fast bacilli. Based on these findings, the patient was started on standard anti‐TB therapy consisting of isoniazid, rifampin, pyrazinamide, and ethambutol (HRZE) for the intensive 2 months phase of treatment.

Additionally, Ultrasonographic evaluation revealed multiple reactive lymph nodes involving the cervical, axillary, and inguinal regions bilaterally. No abdominopelvic lymphadenopathy was detected.

**Figure 4:**
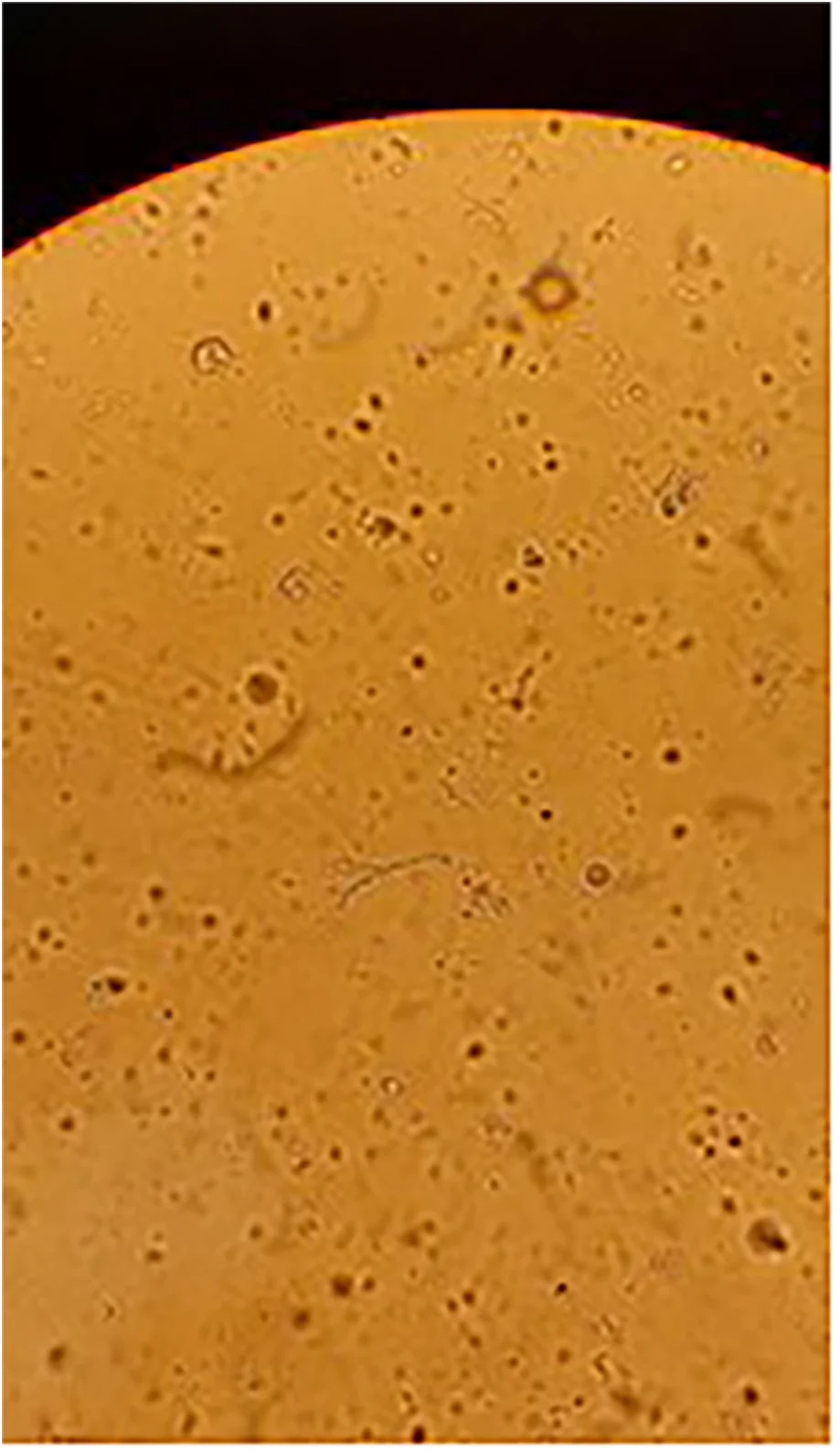
Microscopic examination of the aspirated epidural abscess demonstrating septate hyaline fungal hyphae suggestive of Aspergillus species.

After informed consent was obtained, surgical aspiration of the abscess was performed. Polymerase chain reaction (PCR) analysis of the aspirated material was positive for MTB. Histopathologic examination demonstrated hyaline septate hyphae, findings suggestive of Aspergillus species infection (Figure 4). Following aspiration and initiation of treatment, the patient's neurological status improved, and he regained the ability of ambulate with assistance.

During hospitalization, the patient experienced recurrent episodes of fever and agitation, prompting a sepsis workup. Follow‐up imaging revealed new pulmonary lesions. Pulmonary auscultation demonstrated diminished breath sounds accompanied by expiratory wheezing. Given the low procalcitonin level (0.196 ng/ml), the existing anti‐microbial regimen was continued. Owing to suspicion of CPA, a serum galactomannan assay was performed and yielded a positive result. consequently, liposomal amphotericin B, ceftriaxone, metronidazole, and clindamycin were added to the treatment regimen. In addition, voriconazole was administered at a dose of 200 mg every 12 h.

At presentation, the patient's serum calcium level was elevated (Table 1). On the first day of admission, the serum calcium concentration was 12.08 mg/dl and increased to 13.24 mg/dl within 12 h. Primary hyperparathyroidism, the most common cause of hypercalcemia, was excluded based on laboratory findings, including a parathyroid hormone (PTH) level of 1.66 pg/ml, phosphorus level of 4.26 mg/dl, and albumin level of 3.48 g/dl. Malignancy was also considered in the differential diagnosis; however, computed tomography findings revealed no evidence suggestive of an underlying neoplastic process.

**Table 1:** Baseline blood investigations, the first day of admission.

Laboratory investigations	Values
WBC	7.9
Neut (%)	85.4
Lymph (%)	12.4
MXD (%)	2.2
RBC	4.46
Hb	11.6
Hct	36.4
MCV	81.61
MCH	26.01
MCHC	31.87
Plt	421
ESR‐1 h	83
AST	31
ALT	29
ALP	994
Bili‐T	0.68
Bili‐D	0.42
Ca	12.08
Phos	4.25
Mg	1.86

Abbreviations: ALP, alkaline phosphatase; ALT, alanine aminotransferase; AST, aspartate aminotransferase; Bili‐D, direct bilirubin; Bili‐T, total bilirubin; Ca, calcium; ESR‐1 h, erythrocyte sedimentation rate (1st hour); Hb, hemoglobin; Hct, hematocrit; Lymph, lymphocyte; MCH, mean corpuscular hemoglobin; MCHC, mean corpuscular hemoglobin concentration; MCV, mean corpuscular volume; Mg, magnesium; MXD, mixed cells (monocytes + eosinophils + basophils); Neut, neutrophil; P, phosphorus; Plt, platelet count; RBC, red blood cell; WBC, white blood cell.

Upon admission, the patient reported constipation and myalgia both of which were considered manifestations of hypercalcemia. Electrocardiography demonstrated ST‐segment depression and prominent U waves, findings attributed to hypercalcemia. However, no immediate cardiac intervention was required.

Despite these relatively mild clinical manifestations, the patient's serum calcium concentration continued to rise rapidly, reaching a peak value of 18.01 mg/dl on the eighth day of hospitalization. Given the severity and progressive nature of the hypercalcemia and the risk of life‐threatening complications, therapeutic intervention was initiated.

**Figure 5:**
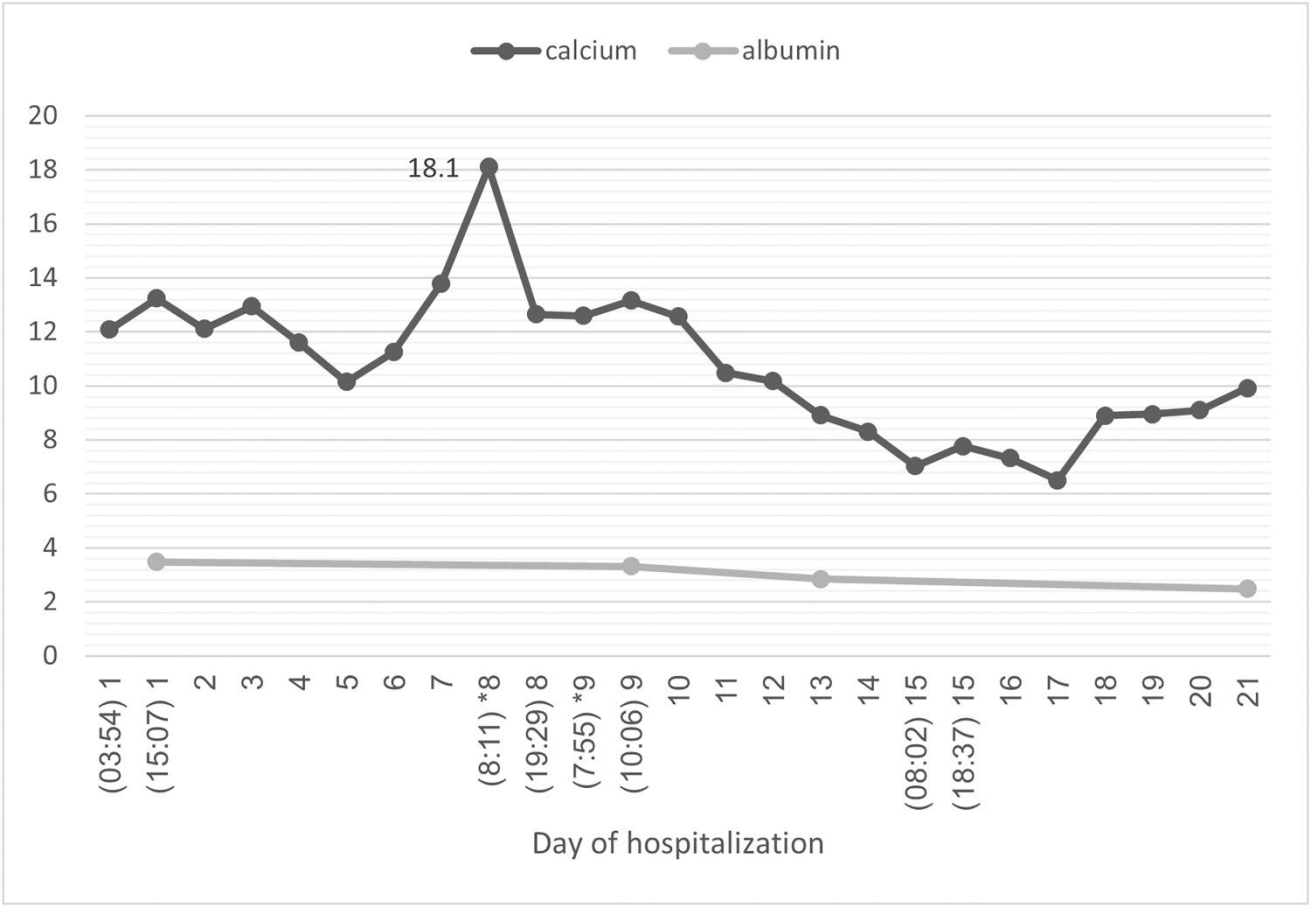
Trends of the patient's serum calcium (mg/dl) and albumin (g/dl) levels during hospitalization. Intravenous pamidrinate (90 mg) was administered on the eighth and ninth days of hospitalization, as indicated by the astrisks (*).

Given the aggressive intravenous (IV) hydration was ineffective in controlling the hypercalcemia (3 liters of normal saline over 24 h followed by 1 liter every 6 h), calcitonin was administered in both injectable and intranasal formulation. To minimize the risk of treatment‐related electrolyte disturbances, only liposomal amphotericin B was continued following consultation with the nephrology team. As the serum calcium level failed to demonstrate a significant decline, pamidronate was administered on two consecutive days. Figure 5 depicts the temporal changes in the patient's serum calcium and albumin levels.

The patient was ultimately discharged with a corrected serum calcium level of 11.12 mg/dl and was advised to continue treatment on an outpatient basis. Close follow‐up was recommended to monitor treatment response and detect potential relapse. However, the patient did not attend any scheduled follow‐up visits, and subsequent clinical outcomes could not be determined.

## 2 Discussion

The present case highlights the diagnostic challenges associated with disseminated TB presenting with predominantly musculoskeletal manifestations. The patient had experienced chronic progressive back pain for several months without a definitive diagnosis or appropriate treatment. Initially, the clinical presentation was nonspecific and could not readily be attributed to a particular underlying condition. However, further diagnostic evaluation revealed pulmonary abnormalities that raised strong suspicion for miliary TB.

The diagnosis was supported by the combination of compatible clinical manifestations, characteristic radiological findings, and three consecutive positive sputum smears for acid‐fast bacilli (AFB). In addition, PCR analysis of the aspirated epidural abscess was positive for MTB. Although mycobacterial culture and GeneXpert testing were not performed, the available evidence was considered sufficient to establish the diagnosis and initiate anti‐TB therapy.

Molecular testing (GeneXpert) was not performed in this case; therefore, definite assessment of drug resistance was not possible. Nevertheless, the favorable clinical response to treatment, together with conversion to negative follow‐up sputum smears, indirectly supports the effectiveness of the prescribed anti‐TB regimen.

A second diagnostic challenge was the possibility of concomitant aspergillosis. Although the serum galactomannan assay yielded a positive result, this finding alone is not specific for pulmonary aspergillosis and may be influenced by several clinical conditions. Moreover, the radiological features were not characteristic of CPA.

However, histopathological examination of the aspirated paravertebral abscess demonstrated septate hyaline fungal hyphae morphologically suggestive Aspergillus species. In the context of clinical deterioration, persistent febrile episodes, and the development of new pulmonary lesions despite anti‐TB therapy, a probable concomitant Aspergillus infection was considered.

Definitive microbiological confirmation through fungal culture or molecular identification was not obtained; therefore, aspergillosis could not be diagnosed with certainty. This represents a limitation of the case.

A possible explanation for the concomitant Aspergillus infection is that chronic TB associated immune dysregulation increased the patient's susceptibility to opportunistic fungal infections [13]. In addition, structural lung damages caused by pulmonary TB may provide a favorable environment for A. fumigatus to colonize and proliferate [13]. Consequently, pulmonary TB is considered as the most important risk factor for CPA [13]. The prevalence of TB‐Aspergillus co‐infection has been reported to be approximately 14.7% in Asia [9]. Despite this relatively high rate of concurrence, routine screening for Aspergillus infection is not commonly incorporated into the diagnostic evaluation of patients with TB.

The patient's initial presentation was dominated by severe neurological deficits consistent with cauda equina syndrome; most likely resulting from compression of the cauda equina nerve roots by the epidural abscess. Surgical decompression and drainage were indicated; however, the patient declined operative intervention. Nevertheless, the initiation of anti‐TB therapy was associated with partial neurological recovery and restoration of assisted ambulation ability.

Among the various aspects of this case, severe hypercalcemia represented the most significant clinical challenge. Overall, hypercalcemia is a relatively common metabolic disorder, with a reported prevalence ranging from 0.2% to 4% among community‐dwelling individuals and hospitalized patients [11, 14]. Its incidence varies according to the population studies, including outpatient, inpatient, and emergency department settings [11].

Malignancy‐associated hypercalcemia and primary hyperparathyroidism account for the majority of causes. Their relative frequencies differ depending on the clinical setting, with malignancy predominating among hospitalized patients and primary hyperparathyroidism being more common in outpatient practice [11]. Consequently, the initial diagnostic approach to hypercalcemia should focus on excluding these two conditions.

Less common causes, including vitamin D intoxication, granulomatous disorders, cosmetic injections, thyrotoxicosis, adrenal insufficiency, milk‐alkali syndrome, medications, familial hypocalciuric hypercalcemia, and immobilization, collectively account for fewer than 10% of causes [14]. Granulomatous diseases may induce hypercalcemia through ectopic production of 1,25 (OH)_2_ D_3_ [11]. Both TB and certain fungal infections are recognized causes of hypercalcemia through this mechanism [11]. in addition, infectious processes may contribute to hypercalcemia through local bone destruction [15].

Therefore, the presence of hypercalcemia in our patient was biologically plausible given the coexistence of disseminated TB, fungal infection, and vertebral destruction. Approximately 88% patients with TB are reported to experience mild, asymptomatic hypercalcemia, which is often detected only through routine laboratory evaluation [1]. Nevertheless, the severity of hypercalcemia was remarkable. in the setting of the mentioned pathologies, the calcium level hardly exceeds 13 mg/dl [1]. In contrast, our patient developed severe hypercalcemia with a peak serum calcium concentration of 18.01 mg/dl, making this presentation highly unusual.

TB‐associated hypercalcemia is generally mild and often resolves with treatment of the underlying infection. Anti‐TB therapy particularly isoniazid and rifampin, has been reported to contribute to normalization of serum calcium levels [11]. Consequently, management is frequently limited to conservative measures while treatment of the underlying disease is initiated. Several studies have suggested that intravenous hydration together with restriction of dietary calcium and vitamin D intake may be sufficient in many cases [1, 15].

The clinical course observed in our patient was markedly different. Despite aggressive intravenous hydration and initiation of anti‐TB therapy, serum calcium levels continued to rise rapidly. Furthermore, calcitonin administration failed to achieve adequate biochemical control, and the serum calcium concentration reached 18.1 on the eighth day of hospitalization. So, treatment with bisphosphonate was initiated.

Notably, a single administration did not produce a satisfactory response. Therefore, intravenous Pamidronate (90 mg) was administered in two consecutive doses. Following treatment, serum calcium levels gradually declined and reached near normal values before discharge.

Severe risk factors for the development of hypercalcemia in patients with TB have been reported, including elevated serum creatinine at admission, renal failure, diuretic use, severe anemia, disseminated TB, diabetes mellitus, hypertension, and human immunodeficiency virus (HIV) infection [1]. Among all, only disseminated TB and mild anemia (hemoglobin approximately 10 g/dl) were present in our patient. HIV status was not assessed.

Evaluation for HIV infection would have been valuable for several clinical and epidemiological reasons. TB and HIV are closely interconnected, as each infection may influence the course and progression of the other through impairment of host immune defenses [16, 17]. accordingly, the world health organization (WHO) strongly recommends HIV testing for all patients diagnosed with tuberculosis [18]. Furthermore, HIV infection is a known risk factor for disseminated and miliary TB [19].

In the present case, the coexistence of a probable Aspergillus infection further raised the possibility of an underlying immunosuppressed state [20]. Consequently, this can be called as another limitation of our study.

A comparable case was reported by Spindle et al. in 1995. Their patient was HIV‐positive and developed an opportunistic fungal infection accompanied by persistent hypercalcemia. Similar to our patient, the hypercalcemia proved difficult to control. However, in this case, serum calcium levels eventually normalized after approximately 4 weeks of intravenous hydration [21].

Our patient presented with disseminated Tb and a probable concomitant Aspergillus infection. Although both TB and fungal infections have been individually associated with hypercalcemia, the contribution of each condition to the severity of hypercalcemia in this case cannot be determined with certainty. Based on available clinical data, it remains unclear whether a synergistic interaction between these infections contributed to the unusually severe and treatment‐resistant hypercalcemia observed in our patient.

### 2.1 Limitations

This case has several limitations. Mycobacterial culture and molecular testing (GeneXpert) were not performed, precluding definitive assessment of drug susceptibility. In addition, although serum galactomannan positivity and histopathologic identification of septate hyaline hyphae in the paravertebral abscess raised susception for a concomitant Aspergillus infection, definitive microbiological confirmation was not obtained. Furthermore, radiological findings were not characteristic of pulmonary aspergillosis. Therefore, the possibility of a concomitant Aspergillus infection should be interpreted with caution.

In addition, the assessment for HIV infection were not performed, which was of necessities in this case.

## Author Contributions

**Negin Parsa:** writing – original draft, writing – review and editing. **Amirpasha Amlelshahbaz:** resources, visualization. **Hamidreza Soltani:** writing – review and editing, supervision, methodology, conceptualization.

## Funding

The authors received no specific funding for this work.

## Ethics Statement

Ethical approval was not required for this case report according to institutional policy. All clinical information was collected and reported in accordance with the principles of the Declaration of Helsinki.

## Consent

Written informed consent for publication could not be obtained because the patient was lost to follow‐up and could not be contacted despite reasonable efforts. All potentially identifiable information has been removed from the article, and the case has been presented in a fully anonymized manner.

## Conflicts of Interest

The authors declare no conflicts of interest.

## Data Availability

The data that support the findings of this study are available from the corresponding author upon reasonable request. All data generated or analyzed during this study are included in this published article. Additional information is available from the corresponding author upon reasonable request.
